# The eye that binds: Feature integration is not disrupted by saccadic eye movements

**DOI:** 10.3758/s13414-019-01873-7

**Published:** 2019-12-05

**Authors:** Josephine Reuther, Ramakrishna Chakravarthi, Amelia R. Hunt

**Affiliations:** University of Aberdeen, Aberdeen, UK

**Keywords:** Attention, Eye movements, Object recognition

## Abstract

Feature integration theory proposes that visual features, such as shape and color, can only be combined into a unified object when spatial attention is directed to their location in retinotopic maps. Eye movements cause dramatic changes on our retinae, and are associated with obligatory shifts in spatial attention. In two experiments, we measured the prevalence of conjunction errors (that is, reporting an object as having an attribute that belonged to another object), for brief stimulus presentation before, during, and after a saccade. Planning and executing a saccade did not itself disrupt feature integration. Motion did disrupt feature integration, leading to an increase in conjunction errors. However, retinal motion of an equal extent but caused by saccadic eye movements is spared this disruption, and showed similar rates of conjunction errors as a condition with static stimuli presented to a static eye. The results suggest that extra-retinal signals are able to compensate for the motion caused by saccadic eye movements, thereby preserving the integrity of objects across saccades and preventing their features from mixing or mis-binding.

**Electronic supplementary material:**

The online version of this article (10.3758/s13414-019-01873-7) contains supplementary material, which is available to authorized users.

## Introduction

We experience a world of unified objects, in which confusion and ambiguity around which features belong to which object seem to be rare. But are they? The classic Feature Integration Theory (FIT; Treisman & Gelade, 1980) posits that attention is required to bind visual features into unified objects. FIT proposes that selective spatial attention gives objects and their composite parts and features continuity over space and time. Until attention is directed to an object, its features are “unbound” and can be interchanged. Treisman and Schmidt (1982) explored this further by documenting these unbound perceptions. They note that in everyday life, even though we attend to a small fraction of the available perceptual information, we do not experience perceptual errors that we could attribute to mis-binding of features. Cognitive framing could help us keep objects and features bound together (e.g., grass is green, sky is blue). For those features which are interchangeable (cars and their colors, people and their glasses), there is nothing to alert us to the fact that we have misperceived them, unless we attend to them, and in attending to them, we correctly bind them. In a series of five now-classic experiments, Treisman & Schmidt explored the prevalence of illusory conjunctions using a dual-task method. The primary task was to report the value of two digits presented on either side of the display. The secondary task was to report the features (position, color, identity, size) of a set of items (digits, letters, or shapes) presented between the two digits. They observed a substantial proportion of conjunction errors, that is, misreporting an object as having features which appeared in the display, but belonged to other objects. The results indicate that conjunction errors may in fact be more common than our experience would lead us to expect.

Several further studies have supported the idea that spatially selective attention plays a critical role in feature integration, and filled out the picture with additional details. Briand and Klein (1987) examined the combined roles of attention in both feature integration (which they refer to as “Treisman’s glue”) and in facilitating detection (which they refer to as “Posner’s beam”, in reference to classic papers by Posner and colleagues (e.g., Posner et al.,, 1980) that show speeded responses to targets appearing in locations indicated by spatial cues). Briand and Klein (1987) cued participants to attend to a particular location using arrows presented either at center, or near the possible target locations in the left or right periphery. Following the cue, a letter-pair would appear in one of these two locations, and the participant had to indicate whether the letter R was present in the pair or not. Importantly, the pair contained features that could either be recombined into an R (P and Q) or they could not (P and B). The cueing effect is the difference in speed to correctly respond to letter-pairs presented in the cued, relative to the opposite, location. Evidence that the attentional “beam” also functions as “glue” would come in the form of larger cueing effects for P and Q pairs than for P and B. They did find this interaction, but only for the peripheral cues, not for the central ones. This suggests a shared association of attention binding and facilitated processing, at least for peripheral cues. Others have examined the role of both spatial and temporal selection in feature binding. Vul and Rich (2010) cued participants to report a single item from spatial and temporal arrays of colored letters. When they increased the noise in the cue signal, they observed a lack of correlation in report errors, meaning that participants were as likely to incorrectly report the letter from one item and the color from another as they were to report the letter/color of a single item. They argue that the visual system samples features with spatial and temporal constraints, and when those constraints are sufficiently tight, binding will be accurate, but when they include features from multiple objects, these are reported in arbitrary combinations.

Given the close relationship between attention orienting and eye movements, a closely related question is whether eye movements have an effect on feature binding. Although attention can shift in the absence of an eye movement, many have argued that eye movements cannot be executed without the involvement of spatial attention (e.g., Deubel & Schneider, 1996; Shepherd et al.,, 1986). This argument is supported by many studies showing that attention is allocated to the goal of a saccade just prior to saccade execution (Born et al., 2014; Hoffman & Subramaniam, 1995; Kowler et al., 1995; Peterson et al., 2004). However, this coupling of attention and saccade target selection may not be obligatory (Belopolsky & Theeuwes, 2009; Henderson, 1992; Remington, 1980; Stelmach et al., 1997; Stigchel & Vries, 2015). To resolve this contradiction, Hunt et al., (2019) have suggested that an obligatory coupling between attention and eye movements may be particular to the typical experiment in which their relationship is measured, that is, when saccades are directed to specified locations in response to the experimenter’s instructions. Deliberate, directed eye movements may necessarily engage spatial attention, but the saccades we spontaneously produce in more natural circumstances may not. Nonetheless, it is clear that attention and eye movements are tightly interconnected and it seems likely that they usually shift in tandem. This has been taken as evidence that selecting a target for a motor action also functions to bind the features in that location into a single unit (Schneider, 1995). This theory suggests that there should be fewer illusory conjunctions at the saccade goal, the presumed site attention is allocated to, relative to other locations that are not the center of attention.

Eye movements may pose an additional challenge for binding features into objects, given that feature maps are widely agreed to be retinotopic (Gardner et al., 2008). Eye movements shift visual information across the retina, consequently also shifting the information’s coordinates in retinotopic feature maps. If attention to aligned locations on retinotopic feature maps is a requirement for binding, one might expect eye movements to interfere with binding, either by mis-aligning or disrupting feature maps, or by using up attentional resources, or both. However, mechanisms exist within the visual system to minimize the potential disruptive effect of eye movements; it is widely agreed that information from the eye movement system is sent to visual areas (e.g., Sperry, 1950), and that this efference copy signal is instrumental in stabilizing perception, despite the massive change across the retina caused by the eye shifting (Wurtz, 2008). The exact nature of this signal and how it is used is the subject of some debate, but one plausible account is that information from the eye-movement system allows attention to be maintained on task-relevant spatial locations when they shift on the retina (Cavanagh et al., 2010). This shift of attention pointers coincides with (or even anticipates) the expected change in the position of important information on the retina associated with each eye movement. This account is consistent with results from neurophysiological (Duhamel Colby and Goldberg, 1992) and behavioral (Jonikaitis et al., 2012; Rolfs et al., 2011) studies. Given that attention is largely agreed to be limited to some small subset of possible objects (Broadbent, 1957), the attentional pointer account of visual stability implies a restricted set of attended (bound) objects could be maintained across eye movements.

Careful measurement of visual perception in laboratory settings has revealed that our perception around the time of eye movements is also subjected to distortions, either in spite of the mechanisms supporting perceptual stability, or because of them. Classic research on localization of brief targets presented during eye movements has found that their perceived position is shifted, an effect known as peri-saccadic displacement (e.g., Honda, 1989, Matin & Pearce, 1965). These shifts were initially observed to be in the direction of the eye movements, but the size of the displacement depended on where in the field they were presented. Later research showed that visual targets presented just before the eyes begin to move are also perceived to be displaced towards the saccade target, even though they are presented to a stable retina (Schlag & Schlag-Rey, 1995), and this has been characterized as a pre-saccadic compression of space (Ross et al., 1997). These compression effects can not only cause a disruption of spatial localization, they have also been shown to cause multiple objects to align, and be seen as only one (Chapman et al., 2007; Ross et al., 1997). There is also evidence that objects begin to be processed as though they were already in their expected retinotopic coordinates, even before the saccade has begun, leading participants to believe the saccade has already occurred (Hunt & Cavanagh, 2009), and causing visual interference between objects in separate locations on the retina (Hunt & Cavanagh, 2011). Together, these results suggest substantial imperfections in our ability to report spatial information accurately for brief objects that appear around the time of the eye movement. Given that accurate feature binding may depend on the alignment of features in visual maps, spatial distortions around the time of an eye movement could lead to mis-binding of features.

While many studies have explored the question of how the perceived location of visual information is affected by eye movements, and how the binding of features is affected by the withdrawal of attention, relatively fewer studies have examined how the perception of non-spatial features is affected by eye movements. Intuition suggests our perception of objects is not disrupted by eye movements, but as we have seen from the research on illusory conjunctions and saccadic displacement noted above, often these effects can only be revealed under carefully controlled laboratory conditions, because normally there are many alternative routes to the perception of objects and space that we can rely on to construct a reasonable estimate of our visual surroundings. Lappe et al., (2006) found that the perception of the distinct colors of objects is preserved even when saccades compress them into the same location. They also showed that when saccadic displacement shifts the perceived location of an object onto an area with the same background color as the object itself, its shape is still visible to the participant. Examining how object features are processed before and after saccades, Golomb et al., (2014) showed that the perceived color of a circle presented after an eye movement was slightly blended, or swapped, with the color of a circle presented in the same retinotopic location before the saccade. This suggests some integration of features presented in the same position on the retina, even though an eye movement has intervened, making this integration an error. In contrast, however, Wijdenes et al., (2015) changed the color of three discs during an eye movement and found integration (mixing) of colors shown before and after the saccade in the same spatiotopic—and not retinotopic—position. To resolve the contradiction, they suggest the continuous presence of the discs throughout the saccade facilitated the correct (that is, from the same object) integration of color information before and after the objects were shifted on the retina by the eye movement. A similar blending of the features of spatiotopically aligned objects has been reported for object contours (Herwig et al., 2015).

In summary, there is clearly a distortion of location information for objects presented briefly before and during saccades. Perception of a nonspatial feature (color) appears to be preserved, however, as does integration of this feature within a single object before and after the eye movements. What is not clear, however, is whether binding of features to objects would be disrupted by eye movements. That is, if multiple objects are presented briefly just before and during an eye movement, during the time where displacement would be expected, will their features nonetheless be correctly bound, or will illusory conjunctions occur? The model of binding proposed by Vul and Rich (2010) suggests the spatial uncertainty introduced by the movement of the retina could interfere with the sampling constraints and lead to increases in illusory conjunctions. Similar predictions arise from the tight relationship that is purported to exist between spatial attention and the execution of eye movements (e.g., Deubel & Schneider, 1996; Shepherd et al.,, 1986).

In the two experiments reported here, we document the prevalence of illusory conjunctions for objects appearing just before, during, and just after eye movement execution, relative to static viewing. Experiment 1 examined illusory conjunctions over both space and time, which allowed us to explore the effect of both the eye movement itself, as well as eye-movement-related shifts in attention, on the prevalence of shape conjunctions (lines of different object are erroneously combined) and color conjunctions (shape and color of different objects are erroneously combined). Based on the literature reviewed above, we expected a modulation of the prevalence of illusory conjunctions related to the execution of an eye movement, namely an increase in locations that were not attended to, and a decrease at the target of the eye movement. The results from this study showed a small elevation in reports of illusory conjunctions, but only for stimuli presented during the saccade and close to central vision. Experiment 2 was designed to target this condition specifically, to confirm whether binding between object identity and color is disrupted during eye movements.

## Experiment 1

The aim of Experiment 1 was to explore the influence attention allocation around and during the execution of an eye movement has on object recognition and feature binding. To this end, we studied two forms of conjunction errors, shape conjunction errors (where line elements of different objects are combined) and color conjunction errors (where the shape of one object was combined with the color of another). Stimuli were presented in three different locations (at the initial fixation, at the saccade target, and in between) and could appear before, during, and after the eye movement. We also included a static condition, with no eye movements. If spatial uncertainty were to increase binding errors, as suggested by Vul and Rich (2010), we should see an increase in illusory conjunctions of both types just before and during the saccade. If the shift of spatial attention to the saccade goal that precedes an eye movement also facilitates binding, as would be expected based on the visual attention model of Schneider (1995), we should also expect to see a reduction in illusory conjunction errors at the saccade target just before and during the eye movement, relative to eccentricity-matched static and post-saccade conditions. The methods and analyses for Experiment 1 were registered on the Open Science Frame work (OSF) and were adhered to unless stated otherwise (https://osf.io/k49mf/).

### Observers

Ten participants (age: 27.4 ± 3.4 years, all right handed, three male) took part in the experiment. Participants were trained observers, recruited from the staff and student body of the University of Aberdeen, and had normal or corrected to normal vision. Participants provided informed consent prior to participation in the study. The study was reviewed and approved by the University of Aberdeen Psychology Ethics Committee.

### Stimuli and materials

**Fig. 1:**
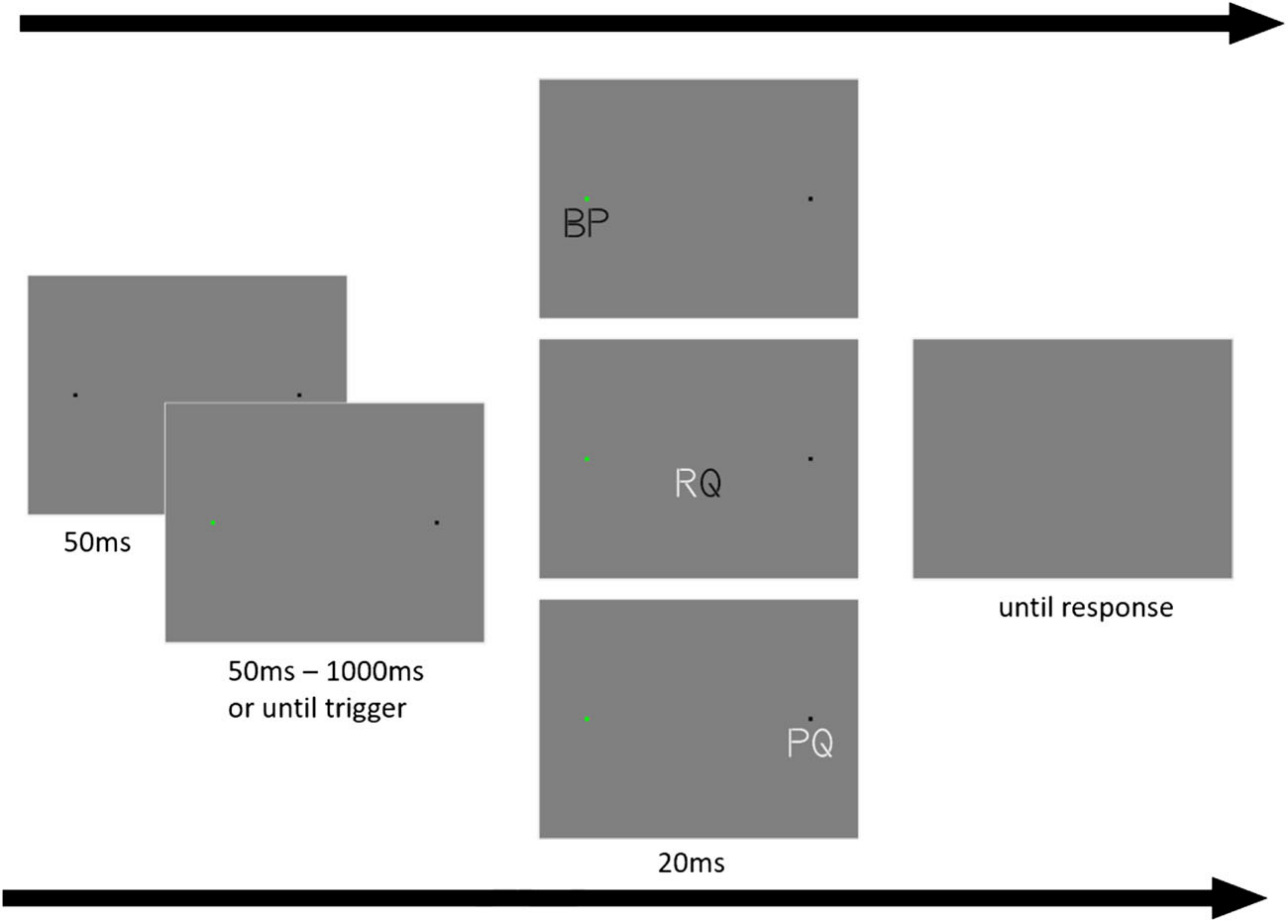
Procedure of Experiment 1: A pair of letters is presented in one of three locations. The initial fixation is the square on the left and the saccade target is the square on the right. Stimulus presentation is triggered in one of four ways: 50 ms after stable fixation of the fixation mark (the pre-saccadic condition); after passing 40% of the distance between fixation mark and the saccade target (peri-saccadic condition); after 50 ms of stable fixation of the saccade target (post-saccadic condition); or after a varying onset time (static condition)

Stimuli were generated in MATLAB using Psychophysics toolbox (Brainard, 1997; Kleiner et al., 2007; Pelli, 1997) as well as Eyelink toolbox extensions (Cornelissen et al., 2002), and were displayed on a 19-inch ViewSonic G90fB CRT-Screen with a frame rate of 100 Hz and a resolution of 1024 x 768 pixels. The screen was turned on at least 10 min before the start of the experiment, which for this model is considered a sufficient warm-up time (Poth and Horstmann, 2017). The viewing distance was set to 55 cm secured by the use of a chin- and forehead-rest. Eye movements were monitored using a desktop-mounted Eyelink 1000 eye tracker (SR Research ltd, Mississauga, Ontario, Canada) with a sampling rate of 1000 Hz. Two small squares were presented along the horizontal meridian, 10^∘^ to the left (fixation-mark) and 10^∘^ to the right (saccade target) from the center of the screen, respectively. The letters B, P, Q, and R served as stimuli in both parts of the first experiment. Example pairs can be seen in Fig. 1. A file containing the stimuli has been deposited on the OSF (https://osf.io/hev38/). Letters were presented in black or white on a mid-grey background. Each of the letters subtended 1.5^∘^ vertically and 1.0^∘^ horizontally, and consisted of two to three constituent parts. Letters were presented in pairs with a center-to-center spacing of 2^∘^. Letter-pairs were displayed 1.75^∘^ below the horizontal meridian, centered in one of three locations: below the fixation mark, below the saccade target, or half way between the fixation mark and the saccade target. The letters that form the pairs either had the same polarity (both white or both black) or the opposite polarity (one white and one black).

### Procedure

The experiment consisted of six sessions that each lasted for approximately 1 h. Each session had two parts. First there was a static part, during which participants were asked to continuously fixate the fixation mark. Second was a dynamic part, during which participants were asked to perform an eye movement from the fixation mark to the saccade target. Each part was split into three blocks, each with a constant stimulus location (described above). During each of the blocks, all twelve possible letter-pairs were presented equally often in each of the four polarity combinations (black-black, white-white, black-white, and white-black). The order of the blocks, and the order of the trials within each block, were randomized. At the beginning of each block, participants were informed in which of the three locations the letter-pair would appear. Each of the blocks was preceded by a nine-point calibration process. Drift correction was carried out at the beginning of each trial.

#### Static part

In total participants performed 864 trials in the static part, 144 trials per session. Each trial started with the presentation of the fixation mark and saccade target. Participants initiated the trial sequence by pressing the space bar while fixating the fixation mark. After 50 ms of stable fixation the fixation mark turned green, and after a varying onset time sampled from a uniform distribution (50 ms:50 ms:1000 ms), the letter-pair was presented for a duration of 20 ms, after which the fixation mark and saccade target disappeared with the letter-pair to indicate the end of the trial presentation sequence. At the end of the trial, participants were asked to report whether they saw an R or not, by pressing one of two buttons (present or absent). If they reported having seen an R, they were also asked to report which polarity the R was presented in by pressing one of three buttons (black, white, or unsure). Trials were aborted when the participant broke fixation during the trial sequence. Fixation was considered to be broken when participants moved their eyes more than 1^∘^ visual angle from the center of the fixation mark. On average 15.3% of the trials were aborted.

#### Dynamic part

In each of the six sessions, the dynamic part consisted of 576 trials, leading to 3456 trials in total. The trial sequence was similar to the sequence described in the static part, apart from the following differences. Firstly, participants were instructed to perform one clean eye movement from the fixation mark to the saccade target once the fixation mark turned green (movement cue). Secondly, stimulus presentation was initiated either by a pre-saccadic trigger (50 ms after movement cue presentation), a peri-saccadic trigger (when the eyes passed the pixel marking 40% of the distance between the fixation mark and the saccade target) or a post-saccadic trigger (50 ms after stable fixation of the saccade target). Lastly, the trial presentation sequence ended after the saccade reached the saccade target (50 ms of stable fixation), and the stimulus was presented. Fixation was considered to be stable if it remained in a 2^∘^ by 2^∘^ square window centered around the fixation mark and the saccade target, respectively.

To assure proper trial execution a number of checks were carried out online. Trials were aborted if participants did not maintain stable fixation for long enough to trigger the movement cue (50 ms within a time window of 5 s). To encourage a direct saccade to the saccade target and to avoid direct stimulus fixation, trials were aborted when participants moved their eyes more than 4^∘^ up, 1^∘^ down or 1^∘^ left from the center of the fixation mark. To encourage continuous eye movements, trials were aborted when the saccade target was not reached within 1s after the presentation of the movement cue. Additionally, to assure that the stimulus was presented to the moving eyes in the peri-saccadic condition, trials with a horizontal velocity of less than two pixels (0.09^∘^) per ms during the duration of the stimulus presentation were excluded from the analysis.

On average 14.3% of the trials were aborted/excluded, fewer than 1% based on a failure to detect stable fixation, 3.2% based an eye movement that was not directed to the saccade target and 2.6% based on not reaching the saccade target in time. Overall 8.4% of all trials were excluded offline because the eyes moved slower than two pixels per ms during stimulus presentation. Note that this exclusion only applies to peri-saccadic trials, which make up one-third of the overall trial number, meaning around 25% of peri-saccadic trials were excluded. The average horizontal velocity for the remaining trials was approximately six pixels (0.27^∘^ ± 0.03^∘^) per ms. Saccade latencies were on average 220 ms measured from the onset of the movement cue.

### Results

**Fig. 2:**
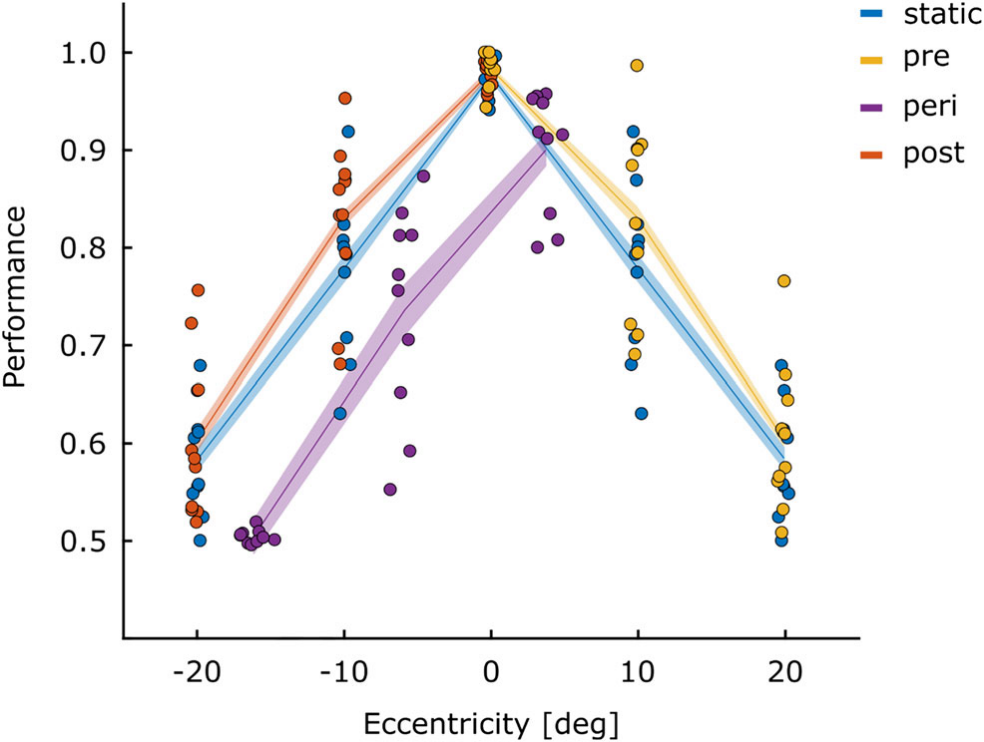
Performance for Experiment 1: Proportion correct for all ten participants is plotted for all four eye movement conditions as a function of stimulus eccentricity. Responses were considered accurate if they correctly reported either presence or the absence of an R. The horizontal placement of the points is based on the average eccentricity for each of the conditions. Data for the static condition (0^∘^, 10^∘^ and 20^∘^) has been mirrored into negative eccentricities (-10^∘^ and -20^∘^) to ease comparison with matching peri- and post conditions. *Lines* indicate the mean and the *shaded areas* indicate ± 1 SEM_*norm*_ (within-subject) in this and all subsequent figures

**Fig. 3:**
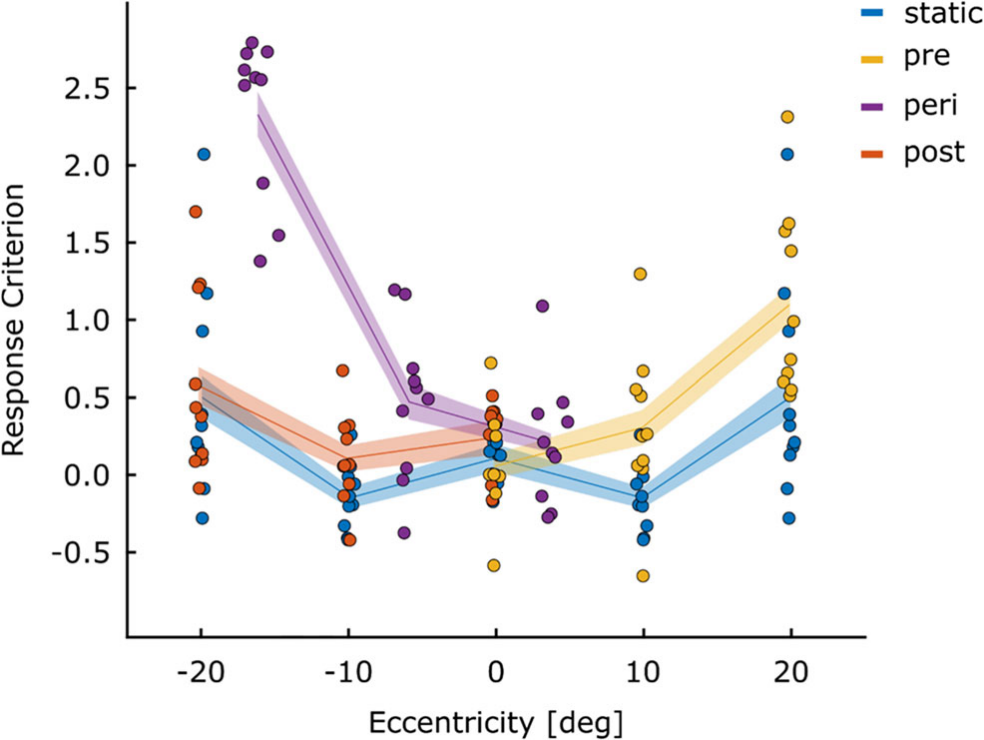
Response criteria for Experiment 1: Response criteria for the R detection response are plotted for each participant for all combinations of eye-movement condition and presentation location. Data for the static condition has been mirrored to the left (-10^∘^ and -20^∘^) for comparison purposes. The closer the values are to zero, the more balanced the response criterion. Positive scores indicate that participants only reported an R when they were relatively certain that they had seen one (conservative). A negative value indicates that participant were more liberal in reporting an R to be present. *Criterion* = − 0.5 ∗ (*Z*[*Hit*] + *Z*[*falsealarm*])

Figures 2 and 3 provide an overview of the participants’ performance and response criteria for each of the conditions resulting from the different stimulus locations and triggers (static, pre-, peri- and post-saccadic). It is clear from Fig. 2 that accuracy was higher with more central presentations. Peri-saccadic presentation lowered performance overall. The response criteria shown in Fig. 3 largely mirror the performance data shown in Fig. 2, with more conservative detection responses under conditions where performance is lower (high eccentricity targets and peri-saccadic display). Statistical analysis of these results can be found in the Supplementary Material. Because the aim of this experiment was to document the prevalence of illusory conjunctions, the remaining results section will address this. For all these analyses, Greenhouse–Geisser corrections were used when the sphericity assumption was violated. Pairwise comparisons were conducted using the Least Significant Difference (LSD) procedure, for which frequentist statistics are accompanied by Bayes-factors (B_01_). Bayes factors were calculated using JASP’s paired samples *t* test functionality.^1^

#### Stimuli presented to a stable retina

We first report the prevalence of illusory conjunctions when letter-pairs were presented to a stable retina (static, pre- and post-saccadic conditions). To investigate the influence of attention allocation before and after the execution of an eye movement, we compared the prevalence with which illusory conjunctions were reported for conditions that are comparable in terms of stimulus eccentricity, but that differed in timing relative to the eye movement. That is, matched for stimulus eccentricity, we compared the results of the pre- and post-saccadic conditions with the static condition.

**Fig. 4:**
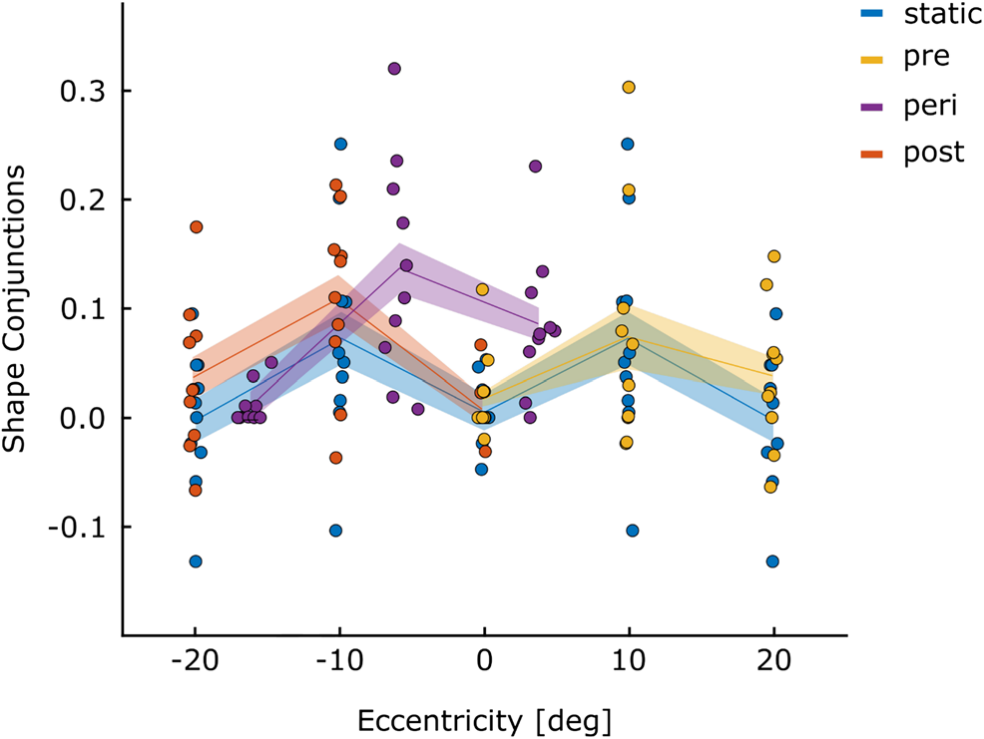
Shape conjunctions: Difference scores for the prevalence of illusory conjunctions for all participants are given for each of the combinations between eye movement condition and stimulus location. Again, data for the static condition has been mirrored to the left (-10^∘^ and -20^∘^) for comparison purposes. Positive values indicate that an R was reported more frequently on trials where the features of the stimulus can be combined to an R (P and Q; illusory conjunction), than when they cannot (P and B, false alarm)

Illusory conjunctions were defined as erroneously reporting the presence of the letter R, specifically on trials where the features of the presented letter-pairs could be mis-combined to perceive an R (i.e., the diagonal line of Q with P). To account for errors due to guessing or motor slips, we subtracted from the proportion of trials with illusory conjunctions, the proportion of trials where an R was falsely reported but could not have been mis-combined from the presented letters (false positive; B and P). Overall, the observed difference scores exceeded 0 (*t*_(9)_ = 4.14;*p* = .001;*B*_01_ = .026), which demonstrates that illusory conjunctions were observed in our setup. This can also be seen in Fig. 4 where values, for the most part, are higher than 0.

To test whether the prevalence of illusory conjunctions is influenced by the timing of the stimulus presentation relative to the execution of an eye movement, differences scores were calculated separately for each of the stimulus presentation locations, eye-movement conditions, and polarity conditions. These scores were then submitted to a 2 (polarity: same or different) by 3 (eye movement condition: static, pre- and post-saccadic) factor repeated measures ANOVAs, individually for each of the stimulus eccentricities (0^∘^, 10^∘^ and 20^∘^), followed up by planned pairwise comparisons between the static condition and the pre-saccadic and post-saccadic condition, respectively.

The location of the letter-pair was blocked and therefore predictable, so that attention could be allocated to the letter-pair location voluntarily. This was done to avoid floor effects, which were observed in a pilot experiment with unpredictable locations. In the pre- and post-saccadic condition, voluntary attention allocation to the target could be superseded by the shift of attention to the saccade target associated with the planned/executed saccade (as originally observed by Shepard, Finlay and Hockey, 1986). Based on these previous research findings showing attention shifts to the saccade target before the saccade, we expected an increase in illusory conjunctions at the fixation location and at the central location in the pre- and post-saccadic conditions (because they are far from the saccade target), when compared to the static condition. Also, if saccadic preparation shifts attention to the saccade target, illusory conjunctions should decrease in the pre-saccadic condition when the stimulus was presented close to the saccade target.

Our results were not in line with any of these predictions. Even though we found a main effect of eye-movement condition at the furthest location ($F_{(2,18)}=5.88; p= .011; {\eta _{p}^{2}}= .395$), at the level of pairwise comparisons, only the difference between the pre-saccadic and the static condition approached significance, yet in the opposite direction of what would have been expected, with more conjunction errors in the pre-saccadic condition than the static. No effects were observed at the other locations (0^∘^: $F_{(2,18)}= .367; p = .698; {\eta _{p}^{2}}= .039$ and 10^∘^:$F_{(2,18)}= .021; p = .979; {\eta _{p}^{2}}= .002$). Polarity had no effect on the formation of shape conjunctions at any eccentricity. All pairwise comparisons can be seen in Table 1.

**Table 1:** Pairwise comparisons: The prevalence of shape conjunctions was compared between the static and the pre-/post-saccadic condition individually for each of the stimulus eccentricities

Dist.	Condition	Polarity	*t*_(9)_	*p*	*B*_01_	Interaction
0^∘^	pre-saccadic	collapsed	− .688	.509	2.654	$F_{(2,18)}= 2.55; p= .106; {\eta _{p}^{2}}= .220$
	post-saccadic		− .191	.853	.100	
10^∘^	pre-saccadic	same	− .178	.862	3.194	$F_{(2,18)}= 3.71; p= 0.45; {\eta _{p}^{2}}= .292$
	post-saccadic		− 1.01	.339	2.134	
	pre-saccadic	opposite	.034	.973	3.236	
	post-saccadic		1.54	.157	1.304	
	pre-saccadic	collapsed	− .042	.973	3.236	
	post-saccadic		− .992	.347	2.164	
20^∘^	pre-saccadic	collapsed	− 2.26	.050	.570	$F_{(2,18)}= 1.77; p= .198; {\eta _{p}^{2}}= .164$
	post-saccadic		− 1.62	.139	1.199	

Note: Since no effect of polarity was observed, data for the same and opposite polarity conditions were collapsed when the interaction between condition and polarity was not significant

**Fig. 5:**
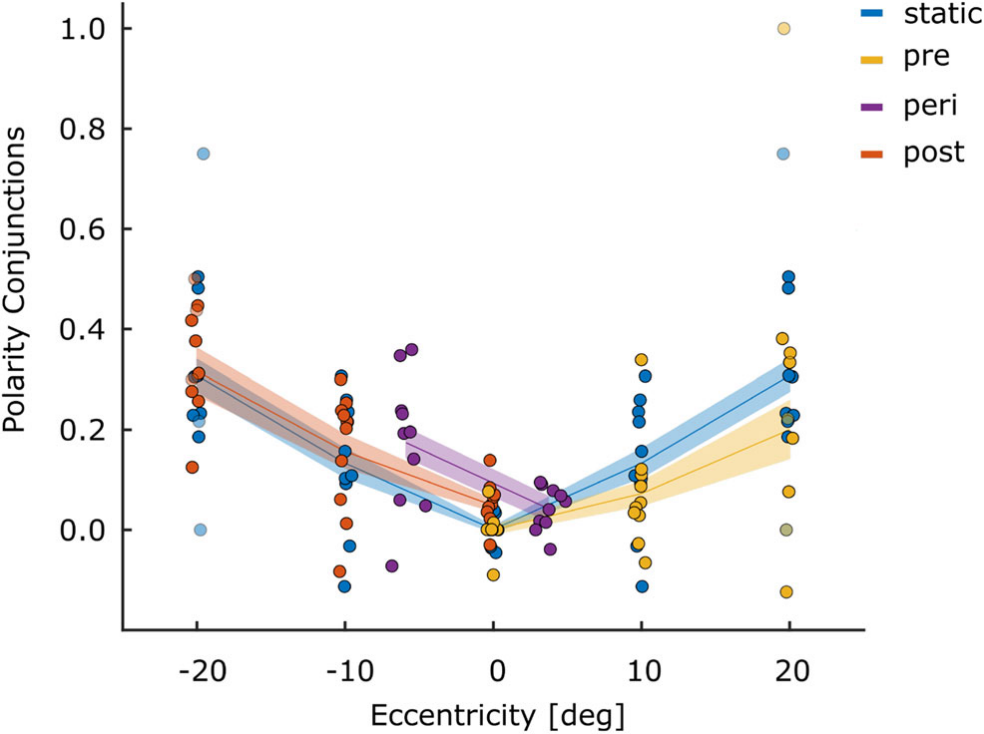
Polarity conjunctions: Difference scores for the prevalence of illusory conjunctions for the trials where an R was reported correctly, but in the wrong polarity. Data for the static condition has been mirrored for comparison purposes. Positive values indicate the predominant report of illusory conjunctions (wrong polarity in different polarity trials), whereas negative values indicate that the predominant report of false alarms (wrong polarity in same polarity trials)

In our setup, illusory conjunctions can also be reflected in the mis-binding between letter identity and polarity. We therefore assessed the proportion of trials during which participants falsely report the polarity of a correctly recognized R (letter R present and reported as such), for trials where polarity swaps are possible (illusory conjunction; different polarity conditions) and for trials where polarity swaps are not possible (error; same polarity condition). From these proportions, we again derived difference scores quantifying the frequency with which illusory conjunctions were formed for each of the stimulus presentation locations, separately for the different eye-movement conditions. Overall, difference scores exceeded 0 (*t*_(9)_ = 7.00;*p* < .001;*B*_01_ = .001), which suggests that these kinds of illusory conjunctions also occurred in our experiment.^2^ This is also obvious in Fig. 5, where values, for the most part, are above 0.

We conducted a three-factor (condition: static, pre- and post-saccadic) repeated measures ANOVA, individually for each of the stimulus eccentricities (0^∘^, 10^∘^ and 20^∘^) to test whether polarity conjunctions are influenced by the timing with which a stimulus is presented in regards to the execution of an eye movement. We found a main effect of eye movement condition at the closest location (*F*_(2,18)_ = 5.79;*p* = .011; ${\eta _{p}^{2}}= .391$), but not at 10^∘^ eccentricity (*F*_(2,18)_ = 1.50; $p= .250; {\eta _{p}^{2}}= .143$). Due to the low number of correctly reported Rs at 20^∘^ we did not assess the frequency of illusory conjunction at this location. At the closest location, illusory conjunctions were more frequent in the post-saccadic, compared to the static and the pre-saccadic condition. All pairwise comparisons can be seen in Table 2. Overall, the results for polarity conjunctions also did not show the reduction of illusory conjunctions that would be expected if planning and executing an eye movement facilitated feature binding at the saccade target location.

**Table 2:** Pairwise comparisons: Prevalence of illusory conjunctions concerning letter polarity was compared between the static and the pre-/post-saccadic condition individually at 0^∘^ and 10^∘^ eccentricity

Distance	Condition	*t*_(9)_	*p*	*B*_01_
0^∘^	pre-saccadic	.035	.973	3.236
	post-saccadic	− 2.85	.019	.270
10^∘^	pre-saccadic	1.47	.175	1.405
	post-saccadic	− .409	.692	3.014

At 20^∘^, the number of trials where the letter R was correctly reported as present was too low for statistical analysis

#### Stimuli presented to the moving eye

Next we explored whether the frequency of conjunction errors associated with stimuli that are perceived during a saccade was higher compared to when stimuli are presented to non-moving eyes. Initially, we had planned to directly compare the results of the peri-saccadic condition to location-matched static pre- and post-saccadic conditions. However, for a high number of trials, the stimulus presentation was initiated later than the targeted eccentricity. This is obvious in the figures, where the results of all conditions are plotted at their actual average presentation location for each of the participants; the purple (peri-saccadic) curves are shifted to the right. To account for the deviation, we randomly sampled trials from the two adjacent locations, and calculated a weighted average to match the location in which the stimulus was actually presented in the peri-saccadic condition. For example, if the stimulus should have been presented at 10^∘^, but was actually presented at 13^∘^ eccentricity against the movement direction, we sampled an equal number of trials from the post-saccadic condition at 20^∘^ and at 10^∘^, and weighted the results from the spatially closer location to be more strongly represented (i.e. to contribute 70%, in this example) in the mean.

To estimate those means, we individually sampled 144 trials from both adjacent locations, assuring an equal number of trials (36) for the combinations of target presence (present or absent) and polarity (same or different). Furthermore, samples were rejected if they contained fewer than 12 trials for both letter combinations of P & Q, as well as B & P. This process was repeated 10,000 times and a weighted average was calculated for each measure of interest and each iteration. To test our hypothesis for each of the three stimulus locations (far against [≈ 16.1^∘^], close against [≈ 5.9^∘^] and close in movement-direction [≈ 3.7^∘^]) the means of the weighted averages over the 10,000 iterations were submitted to 3 factors (condition: peri-saccadic, static and pre-/post-saccadic) repeated measures ANOVAs, followed by pair-wise comparisons.

If the execution of eye movements were to hamper feature integration, we would expect more frequent reports of illusory conjunctions when stimuli are presented during an eye movement (peri-saccadic presentation), than when they are presented to the non-moving eye (static, pre-saccadic and post-saccadic presentation).

Here, a main effect for eye movement condition for the formation of illusory conjunctions (R) was only observed for the furthest distance (≈ 16.1^∘^; $F_{(2,18)}=6.25; p= .009; {\eta _{p}^{2}}= .410$), where fewer illusory conjunctions were reported when the eyes moved away from the stimulus location during stimulus presentation (peri-saccadic condition) compared to when the eye had stopped moving before the stimulus was presented (post-saccadic presentation). No effects were observed when the stimulus eccentricity was smaller (≈ 5.9^∘^: $F_{(2.18)}=2.06; p= .157; {\eta _{p}^{2}}= .186$; ≈ 3.7^∘^$F_{(2,18)}= .467; p= .634; {\eta _{p}^{2}}= .049$). All pairwise comparisons can be seen in Table 3.

**Table 3:** Pairwise comparisons: Prevalence of shape conjunctions was compared between peri-saccadic stimulus presentation and stimulus presentation to the non-moving eye (static, pre- or post-saccadic)

Distance		Condition	*t*_(9)_	*p*	*B*_01_
far	(against movement-direction)	static	− 1.79	.107	.997
		post-saccadic	− 3.02	.014	.216
close	(against movement-direction)	static	1.84	.100	.944
		post-saccadic	.828	.429	2.435
close	(in movement-direction)	static	.969	.358	2.203
		pre-saccadic	.574	.580	2.815

For illusory conjunctions related to the polarity of the letters, we analyzed the data for both close distances (against and in movement-direction). For the far distance the number of correctly reported R’s was too low to warrant statistical testing. For the remaining distances, we observed main effects for presentation condition. When presented against the movement direction ($F_{(2.18)}=5.44; p= .014; {\eta _{p}^{2}}= .377$), we observed more illusory conjunctions when stimuli were presented to the moving eye (peri-saccadic), than when stimuli were presented after the eye movement had been executed (post-saccadic; *t*_(9)_ = 3.09;*p* = .013;*B*_01_ = 5.05). However, no difference was observed when compared to the static condition (*t*_(9)_ = 1.71;*p* = .121;*B*_01_ = .919). When presented in the direction of the movement ($F_{(2,18)}=3.694; p= .045; {\eta _{p}^{2}}= .291$), a difference did not emerge at the level of pairwise comparisons (compared to pre-saccadic: *t*_(9)_ = 1.62;*p* = .139;*B*_01_ = .836; compared to static: *t*_(9)_ = − 1.21;*p* = .258;*B*_01_ = .553).

## Experiment 2

In Experiment 1, an increase in the prevalence of illusory conjunctions was only observed for the binding of shape and polarity, and only when the stimuli were presented during an eye movement in a position close to the current location of the eye. There was no evidence of an effect of pre-saccadic shifts of attention on feature binding, with all eccentricity-matched conditions where the retina was stationary showing roughly equivalent prevalence of illusory conjunctions. The elevation in illusory conjunctions during peri-saccadic presentation requires further experimentation, for two reasons. First, illusory conjunctions clearly vary with eccentricity, and the eccentricity of the presentation in the peri-saccadic condition in Experiment 1 was not precisely matched to the others. To compensate for this, we estimated post-hoc what the illusory conjunction rate would have been for a matched static presentation based on bootstrapped sampling from nearby eccentricities, but ideally the stimuli would have been matched in physical location. Second, illusory conjunctions could be caused by the eye movement itself, or equally by the spatial uncertainty associated with the letters shifting on the retina. In Experiment 2, we addressed both these issues, and focused our investigation on the binding between color and shape for stimuli that were either presented during an eye movement, or to the static eye. We closely matched the presentations for eccentricity and retinal motion by controlling the presentation of the letter-pair in the static condition using the data collected from the same participant in the peri-saccadic condition. Because the individual variation in Experiment 1 was large, we also collected data from a larger sample.

### Observers

Twenty-five participants (aged 24.1 ± 9.7 years, 16 female, nine male, one left handed) took part in Experiment 2. Participants were untrained observers that were recruited and tested as part of a group project. All participants had normal or corrected-to-normal vision and had no known deficiencies in color perception. Participants provided informed consent and the study was reviewed and approved by the University of Aberdeen Psychology Ethics Committee.

### Stimuli and Material

**Fig. 6:**
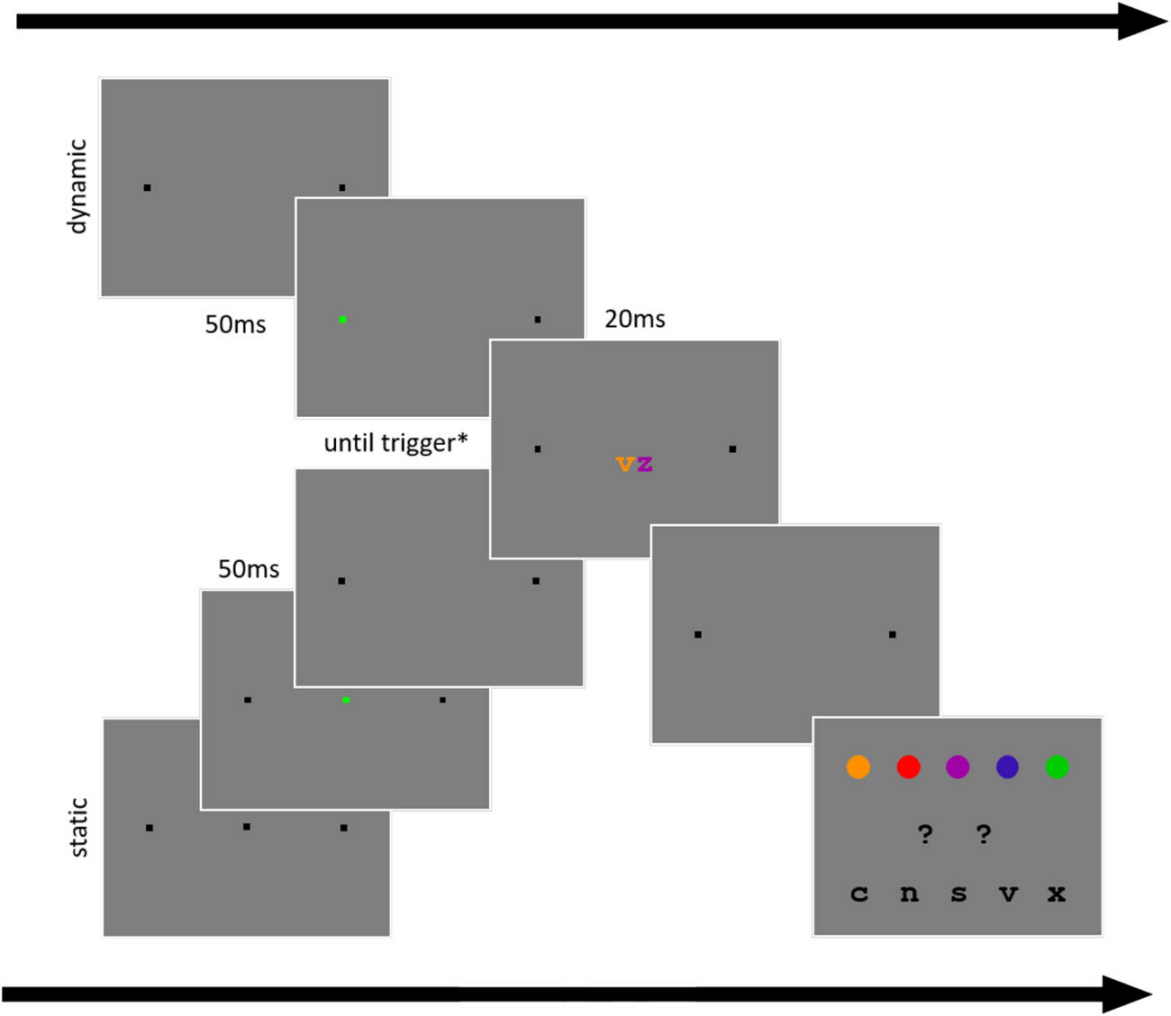
Procedures for the dynamic condition (*top*) and the static conditions (bottom) of Experiment 2: In the dynamic condition participants initially fixated the square on the left until it turned green, and then executed a saccade to the square on the right. The stimulus was triggered after passing 40% of the distance between fixation mark and saccade target (peri-saccadic condition). In the static condition, participants fixated the center square, and continued to fixate the center location when the square turned green and then disappeared. Here the stimulus was presented after an onset time randomly selected from the onset times sampled from the previous dynamic block. Horizontally, the stimulus was presented at center (peri-saccadic) or with an offset from the current fixation position randomly selected from the previous dynamic block (static). Letter-pairs were shown for 20 ms. On half of the trials in the static condition, the letter-pairs moved a comparable distance to the shift caused by the eye movement, and on the other half they were shown in a single location. Participants reported their perception using the mouse selecting a placeholder and then its color and identity using the interface in the rightmost panel

The same stimuli and material as in Experiment 1 were used, with the following exceptions: Instead of the letters B, P, Q and R, the letters c, s, v, x, and z were used as the stimuli.^3^ These letters were presented in 84 pt lower case Courier font in one of 5 colors (orange, red, purple, blue or green)^4^ on a mid-grey background. Letters were presented in pairs, and always with unique colors and identities. Letter-pairs were always presented in the central location. In the static condition an additional small square was presented in the center of the screen. Responses were given using a mouse. The response screen can be seen in Fig. 6.

### Procedure

Participants were presented with two blocked conditions, a dynamic condition and a static condition. During the static condition, two different trial types (described below) were randomly intermixed. The experiment started with a practice part to allow participants to become familiar with the experiment. The practice part consisted of ten static trials (five per trial type) and ten dynamic trials. The practice part was followed by eight blocks alternating between the conditions, starting with a dynamic block. The practice block, as well as each of the following blocks, were preceded by a nine-point calibration. Drift correction was carried out at the beginning of each trial. Participants were instructed to verbalize their percept to reduce the influence of memory decay between stimulus presentation and response selection.

#### Dynamic condition

The trial sequence in the dynamic condition was identical to the peri-saccadic condition in the dynamic part of Experiment 1. That is, participants were instructed to fixate the leftmost small square until it turned green (after 50 ms of stable fixation), and then to execute a saccade to the small square on the right. The letter-pair was presented for 20 ms. Stimulus presentation was initiated when the saccade had passed the trigger (after passing the pixel marking 40% of the distance between the fixation mark and the saccade target). Stable fixation (50 ms) of the saccade target brought up the response screen. Again, fixation was considered to be stable if it remained in a 2^∘^ by 2^∘^ square window centered around the fixation mark and the saccade target, respectively.

To respond, participants were asked to report the color and identity of both letters using the mouse, by selecting each one of the place holders and choosing the color and identity they recalled having been presented in that location. Participants were free to respond in any order and to change their response, but were forced to choose answers that were unique in color and identity. Confirmation of the response (space bar press) concluded a trial. The same trial termination criteria as in Experiment 1 were applied. However, in Experiment 2 horizontal velocity was monitored online and trials with an average velocity of less than 2 pix (0.09^∘^) per ms over an area starting 1^∘^ left from the trigger and ending 5^∘^ deg right of the trigger were aborted. This area includes the entire stimulus region. Each of the four dynamic blocks consisted of 40 trials. An additional trial was added for every aborted trial, leading to 160 valid trials overall.

On average, participants needed to repeat 60 trials to reach 160 valid ones. Of these repetitions, 1.5% were due to a failure to maintain stable fixation at the beginning of the trial, 32.4% due to failing to make a direct eye movement to the saccade target, 28.7% due to not reaching the saccade target in time and 37.4% due to stopping in the region around the stimulus trigger. For the valid trials, the horizontal velocity during stimulus presentation (calculated offline) was approximately 6 pixels (0.28^∘^ ± 0.04) per ms. The average saccade latency was 241 ms measured from the onset of the cue.

#### Static condition

At the beginning of each static trial, participants were presented with three small squares (10^∘^ left, center and 10^∘^ right), and were asked to fixate the center one (see Fig. 6). After successful drift correction, this square turned green to indicate the start of the trial sequence. Fifty milliseconds thereafter, the central square disappeared, but participants were asked keep fixating the location. After a varying onset time, the stimulus was presented for two frames (20 ms), with a varying x- and y-offset (determined based on procedure described below), either for two frames at the same location, or at two different locations. We will refer to these conditions as *static same* and *static different*, respectively. The response screen was presented 100 ms after stimulus-offset.

The stimulus onset delay, as well as the stimulus locations, were sampled trial-wise from the distribution recorded during the preceding dynamic block. That is, values controlling the time and location of stimulus presentation stemmed from the same randomly sampled trial. This was done to introduce a similar amount of uncertainty about the stimulus presentation time and the stimulus location as had been present in the eye movement block. When the stimulus was presented in two different locations, the x- and y-offsets matched the distance between the position of the eye and the position of the stimulus during stimulus-onset (frame 1) and stimulus-offset (frame 2) of the sampled dynamic trial. When the stimulus was presented in the same location, the x- and y-offset matched the average of the distance between the eye and the stimulus during onset and offset of the sampled dynamic trial for both frames.

### Results

**Fig. 7:**
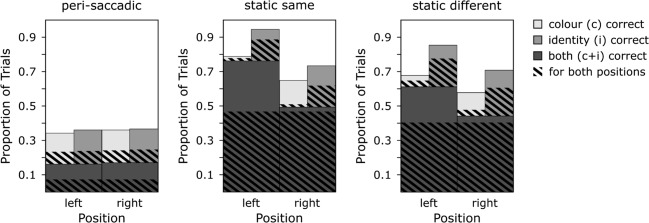
Overview of correct and error trials in Experiment 2. The three panels show the data for the peri-saccadic (*left*) and the two static conditions (static same: *middle* and static different: *right*). The static conditions differed in that for two successive frames the stimulus was either presented in two different locations in eccentricities similar to the those during stimulus-onset (frame 1) and stimulus-offset (frame 2) of the dynamic condition (*mid-grey*) or twice at the same location similar to the average of those during stimulus-on and –offset (*right*). The *stacked bars* show the proportion of trials where both identity and color were reported correctly (*dark-grey*), the trials were only the color was reported correctly (*light-grey*), and trials were only the identity was reported correctly (*mid tone*). The striped overlay indicates the proportion of trials for which identity and/or color were reported correctly for both targets

Figure 7 provides an overview of the performance data for Experiment 2, for each of the conditions (peri-saccadic, static same and static different) separate for both letter positions (left and right). It is clear from this figure that accuracy was higher overall when the eyes remained stationary relative to when they moved. As in Experiment 1, the focus here is on the prevalence of illusory conjunctions in these conditions, so the analysis of this overall performance data is reported in the Supplementary Material.

**Fig. 8:**
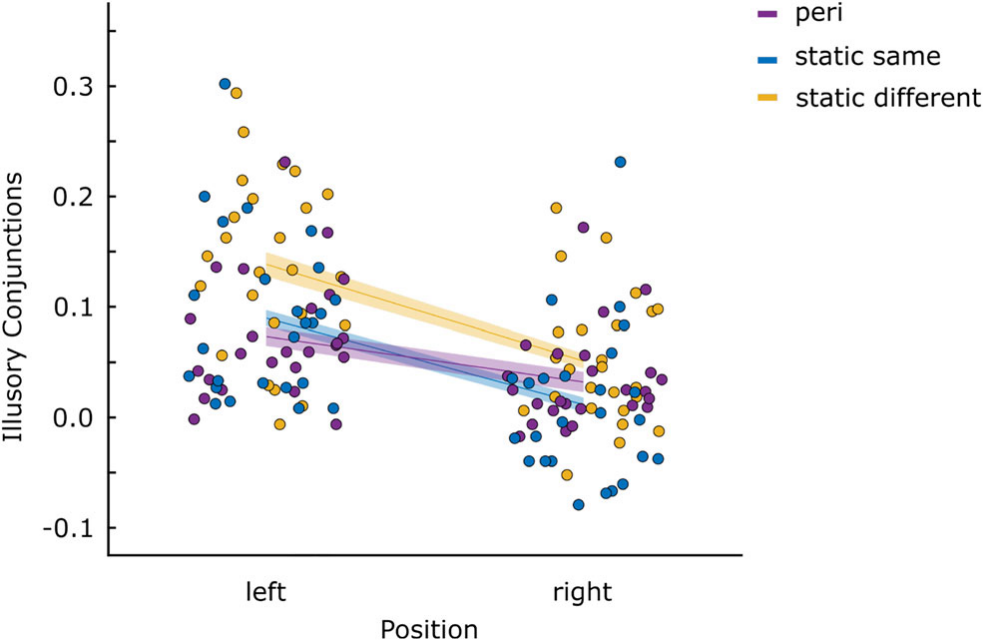
Illusory conjunctions: Difference scores depicting the prevalence of illusory conjunctions in Experiment 2, separately for both target positions and all eye movement conditions. Each *dot* represents the score of one participant in the respective condition. The *lines* represent the mean per condition. *Shaded areas* indicate ± 1 *SEM*_*norm*_ (within subject)

To assess if the frequency of illusory conjunctions differs between the condition where the letter-pair was presented during an eye movement and the conditions where the pair was presented to the static eye, we assessed the proportion of trials were participants reported either the color or the identity of the letter incorrectly, but not both (error). We again calculated a difference score between the proportion of trials with illusory conjunctions (incorrectly attributing a color or identity to one location that was presented in the other) and the proportion of false positives (incorrectly reporting a color or identity that was not presented). Before subtraction, these values were corrected for their respective level of guessing by chance (0.25 and 0.75). The resulting difference scores are plotted in Fig. 8. A positive difference indicates that the number of illusory conjunctions exceeds the number of occasions where an illusory conjunction was reported by chance while a negative difference score indicates that more frequently what appears to be an illusory conjunction is the result of guessing the erroneously reported attribute. Overall, difference scores again exceeded 0 (*t*_(24)_ = 6.430;*p* < .001;*B*_01_ < .001) suggesting that we obtained illusory conjunctions using these stimuli. This can also be seen in Fig. 8, where for the most part, values are higher than 0. If there is an increase in illusory conjunctions related to the execution of an eye movement per se, we would expect a higher difference score for peri-saccadic stimulus presentation compared to stimulus presentation to the static eye. If the increase is related to the spatial uncertainty created by retinal motion, we should see similar illusory conjunction prevalence for the peri-saccadic condition as we do for the static condition with two locations. Inconsistent with either of these patterns, it is clear from Fig. 8 that the condition with the highest prevalence of illusory conjunctions is the static condition with two stimulus presentation locations.

The difference scores were submitted to a 2 (target position: left and right) by 3 (trial-type: dynamic, static same and static different) factors repeated measures ANOVA. We observed a main effect for letter position ($F_{1,24} = 82.3, p < .001; {\eta _{p}^{2}} = .774$), where illusory conjunctions were more frequently reported for the left letter, compared to the right letter. The ANOVA also confirmed an effect of trial-type ($F_{2,48} = 11.6; p < .001; {\eta _{p}^{2}} = .327$) and an interaction between letter position and trial-type ($F_{2,48} = 8.92; p = .001; {\eta _{p}^{2}} = .271$). We again followed this up by pairwise comparisons between the different trial types for both of the letter-positions individually (Table 4). For both letters we did not find a difference between the dynamic condition and the static condition where the stimulus was presented for two frames in one location (static same). The only difference between stimulus presentation to the moving eye and stimulus presentation to the static eye was found for the left letter, where fewer illusory conjunctions were reported in the dynamic condition compared to the static condition where the stimulus was presented in two different locations for 1 frame respectively (static different).

**Table 4:** Pairwise comparisons: The prevalence of illusory conjunctions was compared between peri-saccadic stimulus presentation and stimulus presentation to the static eye

Letter position	Comparisons	*t*_(24)_	*p*	*B*_01_
left	dynamic - static same	− 1.47	.153	1.823
	dynamic - static different	− 4.04	< .001	.015
	static same - static different	− 4.05	< .001	.015
right	dynamic - static same	1.95	.063	.934
	dynamic - static different	− 1.67	.109	1.420
	static same - static different	− 4.74	< .001	.003

In the static trials, stimuli were either presented for two frames in differing locations (static different) or in the same location (static same)

## Discussion

The current study examined whether planning and executing eye movements interferes with feature integration, as measured by the prevalence of conjunction errors. The results of the two experiments demonstrate that they do not. Externally generated retinal motion does increase conjunction errors, but motion of the same extent caused by an eye movement shows similar levels of conjunction errors as stationary targets shown to a stable retina.

The act of making a saccade did not have a consistent effect on the prevalence of illusory conjunctions; the letter-pairs appearing on a stable retina just before or after the eye movement showed similar rates of illusory conjunctions relative to the eccentricity-matched conditions with no eye movement at all. The lack of any pre-saccadic changes in perception accuracy or misbinding of features may seem particularly surprising, in light of the large number of studies showing that attention tends to be allocated to saccade targets just before the eyes move (e.g., Born et al.,, 2014; Deubel & Schneider, 1996; Hoffman & Subramaniam, 1995; Kowler et al.,, 1995; Shepherd et al.,, 1986). There are a few differences in the paradigm we used compared to these earlier experiments, which might account for this discrepancy. First, the stimulus location was blocked in Experiment 1, enabling attention to be allocated to a single location throughout the block, which may have diminished any effect of saccade planning. However, many studies have found effects of saccades despite having predictable target locations (Baldauf & Deubel, 2008; Shepherd et al., 1986). Second, another potentially important feature of our experiment was that the letter stimuli were large (1.5 x 1 degree) and presented below, rather than at, the saccade target. Attention may have been allocated to the saccade target itself, and the benefits of this focus might not have spread to the letter-pair below. Finally, and importantly, Briand & Klein 1987showed that focused spatial attention facilitated performance only when attention had been summoned by a spatial exogenous cue, but not when it was endogenously allocated following a central cue. In this experiment, we directed rightward saccades by changing the color of the left fixation point, meaning no cue was presented in the peripheral location to which the eye movement would be directed. Participants would have had to endogenously allocate their attention to the saccade target. Our results therefore do not directly contradict existing results, while also suggesting that the simple act of making a saccade does not have appreciable effect on feature integration. This could be because saccade planning and execution does not require much attention, with sufficient resources remaining for feature integration. Alternatively, attention may not be required for feature integration. Our results are not, on their own, sufficient to resolve this question.

An intriguing, but tentative, finding from Experiment 1 was the small increase in illusory conjunctions for letter-pairs presented during eye movements relative to letter-pairs presented to the static eye. This effect was isolated to stimuli presented near central vision, but perception of features during saccades at the more distant locations was too poor to reliably measure illusory conjunctions, so the spatial extent of the effect in this experiment should be interpreted with caution. Moreover, direct comparison between the peri-saccadic condition and other conditions was confounded by differences in retinal location and retinal motion. This was the reason we chose to focus on this comparison in Experiment 2, by controlling these factors in a shorter experiment with more participants. When we more carefully controlled retinal position and motion on the retina, we no longer observed elevated illusory conjunctions specific to the peri-saccadic condition, so we can conclude that this aspect of the results of Experiment 1 were due to these factors rather than the eye movement itself.

The striking result from Experiment 2 was that the object pair presented to the static eye (matching the timing, position, and extent of motion of the peri-saccadic condition) produced an increase in the incidence of illusory conjunctions relative to the peri-saccadic condition. The peri-saccadic condition results were similar to the static condition where the stimuli were matched in spatial eccentricity and temporal uncertainty, but only shown in one location. In other words, retinal motion is disruptive to feature integration (consistent with Vul & Rich, 2010), but stimuli presented during saccades are spared this disruption. While the spatial separation of the two presentations was matched across the eye movement and static-different conditions, the former was a single presentation that moved continuously across the retina, while the latter involved presenting the letter-pair in two discrete locations. This discrete presentation might increase masking effects, which could account for our results. To check this, we examined results from the static-different condition separated by whether the two presentations overlapped (where masking should be observed) compared to when the two presentations were spatially separated (where masking should not be observed). To give an example, for two letters, C and V presented for 10 ms in one location followed by a 10 ms presentation in an adjacent location, the distance between the trailing V of the first presentation and the leading C of the second had to be less than that between C and V within any given frame to be considered overlapping. Defined this way, overlap occurred in less than 30% of the trials. Furthermore, if anything, this overlap led to a reduction in illusory conjunctions: For the 30% of the trials with overlap, only 11.9% of the trials contained illusory conjunctions for the left and 2.1% for the right letter, compared to 13.8% and 5.1% when all trials were considered.

In our study, the stimuli in the peri-saccadic condition were presented during what is traditionally considered the ‘saccadic suppression’ phase (Ibbotson and Krekelberg, 2011). It has been extensively documented that stimuli presented to the saccading retina are either not observed or weakly perceived (Erdmann and Dodge, 1898; Volkmann, 1986; Wurtz, 2008; Ibbotson and Krekelberg, 2011). Although retinal sources like masking or smearing are thought to contribute to this suppression, it has been argued that extra-retinal sources, such as corollary discharge from neurons that plan eye movements, also play a role. For example, objects that are presented for a very brief duration during a saccade, thus avoiding retinal smear, can also be suppressed (Mitrani & Yakimoff, 1970). On the other hand, previous studies have noted that chromatic stimuli, such as the colored letters in our study, suffer less suppression than achromatic stimuli (Burr & Morrone, 1996). Similarly, there is evidence that the ability to identify high-contrast letters can be well above chance during saccades (Hunt et al., 2008; Uttal & Smith, 1968). We found that performance was lower in the peri-saccadic condition than in the other non-movement (pre-saccadic, post-saccadic or static) conditions, demonstrating suppression of letter identification during saccades. Nevertheless, we did not find any differences in illusory conjunctions in the peri-saccadic condition relative to the matched conditions, indicating that although overall identification might have been reduced, feature integration of the unsuppressed objects during eye movements is comparable to that when eyes are stationary. Taken together, even if one or more suppressive mechanisms are at play during saccades, it is unlikely that they play a role in feature integration.

In smooth pursuit eye movements, there is a reduction in the perceived smear of physically stationary objects that move across the retina relative to objects moving across the retina at a matched rate while the eyes are stationary (Bedell & Lott, 1996). These authors suggest that an extra-retinal signal suppresses image smear when the eyes are moving. Bedell and Yang (2001) later reported a similar suppression of smear for targets flashed during a saccade, relative to a condition with matched retinal motion but a stationary fixation. Possibly, the mechanisms that suppress the perception of smear also allow for the correct binding of features relative to externally-generated retinal motion. A parallel observation was made by Krüger and Hunt (2013), who observed that inhibition of previously-attended targets is disrupted by predictable external motion, but not by saccadic eye movements. They suggest that the efferent signal from the oculomotor system rapidly and automatically maintains attention in spatiotopic coordinates. In this sense, the saccade can even be suggested to promote feature integration, rather than disrupt it.

It is interesting that saccades can systematically distort the perceived location of briefly presented stimuli and yet not disrupt the binding of features within objects. This result is consistent with the findings of Lappe et al., (2006) showing that the perception of the color of individual objects is preserved even when the objects themselves have been perceived to be spatially compressed towards the saccade target, and that the saccadic displacement of an object onto a field of the same color does not make that object less detectable. To explain their findings, they suggested that localization must be a parallel, or later, process than feature processing. In the case of our results, showing no disruption in feature binding suggests that this process may occur in parallel with localization. This seems inconsistent with FIT, which suggests that directing attention to the shared retinal location of features is a pre-requisite for correctly binding them into a single object. It is possible that attention can be rapidly and correctly allocated to brief targets during a saccade just as well as when the retina is stationary, but the peri-saccadic targets would nonetheless be mislocalized. This might suggest that attention uses a different coordinate system than the one which we experience and rely on to report a location, or perhaps that the shift of attention to track the object as it moves on the retina distorts the experience of the object’s location. It is also important to note that we did not measure saccadic displacement in our experiments, although our subjective experience when piloting the experiment was consistent with saccadic displacement having occurred.

In addition to distorting perceived spatial positions of objects, saccadic eye movements also demand attentional resources. The core assertion of the feature integration theory, that focal attention is necessary to correctly bind the features of an object, is challenged by the absence of any disruption of binding associated with planning and executing and eye movement in our experiments. This is a difficult assertion to disprove. In real world scenarios, binding errors might be corrected by knowledge (e.g. Treisman and Schmidt 1982). However, in a lab setting knowledge about the likelihood of certain feature combinations is often unavailable, and still, correct object feature binding is maintained under attentionally-demanding conditions (Cohen & Ivry 1989, current study). This might be the result of spare attentional capacity; perhaps binding only requires a small amount of attention, so a secondary task that does not utilize attention to full capacity does not provide a clear test of the theory. As considered earlier, in our study, eye movement planning and execution might not occupy attention completely or might allow the refocusing of attention to the stimulus. A different explanation that might explain our data was brought forward by Cohen and Ivry (1989). They suggested that misbinding errors are the result of objects that fall within the same area. This area is either analogous to the size of the attentional focus, where illusory conjunctions are observed when the attention focus is not precise enough to zoom in on a single object, or analogous to the size of the perceptive field, where especially in extra foveal areas small enough perceptive fields are unavailable, thus several objects would fall within the same perceptive field. Even though Cohen and Ivry (1989) were also not able to differentiate between the different possibilities; they suggest that rather than being free floating until captured by focal attention, features might retain some crude location information originating from the neurons in whose receptive fields they fall in, where the formation of illusory conjunctions depends on inter-object spacing.

This debate of attentional focus versus perceptive field size as a cause for illusory conjunctions is reminiscent of the attentional focus versus integration field size debate in visual crowding (Intriligator & Cavanagh, 2001; Pelli et al., 2004). Visual crowding is the deterioration in object recognition in the presence of nearby flankers. This inability to recognize has been argued to be due to a limitation in the resolution of selective attention, where if more than one object falls within the attentional focus, they are bound together leading to a difficulty in recognition (He et al., 1996; Intriligator & Cavanagh, 2001). In contrast, others argued that there are fixed pooling or integration regions where features that fall within them are bound together. When multiple objects fall within one such integration field this leads to misbinding of object features, which causes crowding. Attention plays no role in this latter explanation. Further, Pelli et al., 2004 argued that several previous findings that demonstrated illusory conjunctions are in fact a result of visual crowding: closely spaced features are misbound and this (mis)binding does not require attention. Although eye movements were the focus of our study, our results leave room for the interpretation that binding can occur in the absence of attention, given that binding appears to be modulated by eccentricity and that it was not impaired–and even improved–by an attentionally demanding secondary task.

A final note about our results is that we do observe illusory conjunctions in this experiment. We tested feature integration in three different ways: mis-reporting an R when a P and Q had been presented, mis-binding of an R with the polarity of the distractor letter, and conjunction errors during free report of the identity and color of two letters. In all cases, individual differences were large, but overall the majority of participants had a rate of conjunction errors (minus non-conjunction errors) that was greater than 0. An additional important observation from Experiment 2 is that conjunction error rates were higher for the left position than the right. Participants tended to report the left position first, so their overall accuracy and confidence in their reports of this letter were higher than for the right one. The fact that conjunction error rates were also higher for the left position suggests these errors are likely due to true mis-perceptions, rather than guessing under uncertainty. Our results are therefore consistent with the claims of Treisman and Schmidt (1982) that illusory conjunctions are a more common aspect of our perception than experience would suggest.

*The data for both experiments, as well as a file containing the stimuli of Experiment 1 are available at*https://osf.io/k49mf/, *where Experiment 1 was also preregistered.*

## Electronic supplementary material

Below is the link to the electronic supplementary material.

(TEX 9.84 KB)

## Footnotes

1 Bayes factor results can be considered as anecdotal evidence for H_0_ when B_01_ is between 1 and 3, moderate between 3 and 10, and strong evidence for values above 10. Results below 1 provide evidence for H_1_, which is considered to be anecdotal between 1 and 0.33, moderate between 0.33 and 0.1, and strong for values below 0.1. A B_01_ value of 1 provides evidence neither for H_0_ nor for H_1_.

2 Only data for the later analyzed locations (0^∘^ and 10^∘^) were included. For the location at 20^∘^ eccentricity, the number of trials where polarity binding errors could be assessed was too low.

3 Letters and font were chosen based on their low confusability scores (Bernard & Chung, 2011).

4 RGB and luminance values for the colors of the letters. orange: [255, 145, 1], 51.313 *c**d*/*m*^2^; red: [255, 1, 1], 24.263 *c**d*/*m*^2^; purple: [160, 1, 166], 18.087 *c**d*/*m*^2^; blue: [59, 20, 175], 12.793 *c**d*/*m*^2^; and green: [1, 204, 1], 50.577 *c**d*/*m*^2^
